# Cerebellar and basal ganglia motor network predicts trait depression and hyperactivity

**DOI:** 10.3389/fnbeh.2022.953303

**Published:** 2022-09-16

**Authors:** T. Bryan Jackson, Jessica A. Bernard

**Affiliations:** ^1^Department of Psychological and Brain Sciences, Texas A&M University, College Station, TX, United States; ^2^Texas A&M Institute for Neuroscience, Texas A&M University, College Station, TX, United States

**Keywords:** Human Connectome Project, cerebellum, basal ganglia, depression, hyperactivity

## Abstract

In the human brain, the cerebellum (CB) and basal ganglia (BG) are implicated in cognition-, emotion-, and motor-related cortical processes and are highly interconnected, both to cortical regions *via* separate, trans-thalamic pathways and to each other *via* subcortical disynaptic pathways. We previously demonstrated a distinction between cognitive and motor CB-BG networks (CCBN, MCBN, respectively) as it relates to cortical network integration in healthy young adults, suggesting the subcortical networks separately support cortical networks. The CB and BG are also implicated in the pathophysiology of schizophrenia, Parkinson's, and compulsive behavior; thus, integration within subcortical CB-BG networks may be related to transdiagnostic symptomology. Here, we asked whether CCBN or MCBN integration predicted Achenbach Self-Report scores for anxiety, depression, intrusive thoughts, hyperactivity and inactivity, and cognitive performance in a community sample of young adults. We computed global efficiency for each CB-BG network and 7 canonical resting-state networks for all right-handed participants in the Human Connectome Project 1200 release with a complete set of preprocessed resting-state functional MRI data (*N* = 783). We used multivariate regression to control for substance abuse and age, and permutation testing with exchangeability blocks to control for family relationships. MCBN integration negatively predicted depression and hyperactivity, and positively predicted cortical network integration. CCBN integration predicted cortical network integration (except for the emotional network) and marginally predicted a positive relationship with hyperactivity, indicating a potential dichotomy between cognitive and motor CB-BG networks and hyperactivity. These results highlight the importance of CB-BG interactions as they relate to motivation and symptoms of depression.

## Introduction

The cerebellum (CB) and basal ganglia (BG) are each cytoarchitectonically unique and thought to be foundational to cortical processing, each supporting cognition with specific computations associated with predictive learning (Flanagan and Wing, 1997; Morton and Bastian, 2006; Wolpert et al., 2011) and reinforcement learning (Schultz et al., 1998, 2000; Cohen and Frank, 2009; Ito and Doya, 2011), respectively. Previous work by our lab found a highly-connected resting-state CB-BG network in healthy young adults (Hausman et al., 2019) and a follow-up study using graph theory metrics confirmed distinct motor and cognitive functional networks that were differentially related to cortical functional networks (Jackson and Bernard, 2022). Notably, the CB and BG have each been separately implicated in multiple psychiatric and neurological disorders (e.g., depression, bipolar disorder, psychosis; reviewed in Ring, 2002; Hoppenbrouwers et al., 2008; Macpherson and Hikida, 2019). For example, differently shaped BG regions are found in drug-naïve bipolar disorder sufferers (Hwang et al., 2006) and structural alterations and degeneration of the cerebellum is related to a general liability for mental illness including psychosis and depression (Liszewski et al., 2004; Romer et al., 2018). Additionally, altered CB-cortical functional connectivity (FC) is found in geriatric depression (Alalade et al., 2011) and altered BG-cortical FC in obsessive-compulsive disorder (OCD; Harrison et al., 2009). Research has also linked receptor expression to symptomology. CB GABA (Fatemi et al., 2013) and striatal dopamine (Jauhar et al., 2017) differentiated those with schizophrenia and bipolar disorder from healthy controls. Striatal serotonin also predicted depression symptom severity (Cannon et al., 2007). Here, we are interested in determining whether there is a relationship between cognitive CB-BG (CCBN) or motor CB-BG (MCBN) network FC measures and scores on a series of cognitive tasks and self-reported psychiatric measures in a community sample of healthy young adults.

Though the CB has historically been associated with motor function, the last few decades have highlighted CB contributions to cortical networks and a wide variety behavioral domains including cognition, emotional regulation, and attentional control (reviewed in Stoodley, 2012; Stoodley et al., 2012; Bostan and Strick, 2018; King et al., 2019). Likewise, the BG, a group of mid-brain nuclei including the globus pallidus, substantia nigra, dorsal and ventral striatum (putamen and caudate), subthalamic nucleus, and subdivisions therein, were also historically associated with motor behaviors and function. The theory has since evolved to encapsulate motivation and reinforcement learning across domains (Schmidt et al., 2008; Sgambato-Faure et al., 2016; Bostan and Strick, 2018).

The uniformity of the cerebellar cytoarchitecture has been taken to suggest that the CB performs a similar type of calculation across domains, potentially acting as a modulator for larger networks (Schmahmann, 1991; Ito, 1993, 2008; Ramnani, 2006; Koziol et al., 2014), though in more recent years this idea has been questioned (Diedrichsen et al., 2019). The closed-loop trans-thalamic circuits providing connections to multiple distinct brain regions allow for contributions in many domains. Given the many and varied functional contributions of the CB, it is perhaps not surprising that CB dysfunction is implicated in, and associated with symptoms of many different psychiatric and neurological disorders (Shakiba, 2014). Lesions in the posterior CB, a subregion anatomically and functionally associated with non-motor function in both humans and non-human primates (Dum and Strick, 2003; Kelly and Strick, 2003; Strick et al., 2009; Bernard et al., 2012; Guell et al., 2018; Ren et al., 2019), are linked to cerebellar cognitive affective syndrome, named for its wide-varying deficits in executive function and working memory, visuospatial and verbal memory, affect, and language generation (Schmahmann and Sherman, 1998; Hoche et al., 2018). Further, differences in CB anatomy, CB functional activation, or functional connectivity are associated with many disorders including post-traumatic stress disorder (Baldaçara et al., 2012; Holmes et al., 2018; Rabellino et al., 2018), anxiety (Liu et al., 2015; Doruyter et al., 2016; Li et al., 2016), obsessive-compulsive disorder (OCD; Pujol et al., 2004; Xu et al., 2019), depression (Alalade et al., 2011; Ma et al., 2013; Lai and Wu, 2016; Zhu et al., 2020), schizophrenia and psychosis-related disorders (Meltzer and Stahl, 1976; Collin et al., 2011; Liu et al., 2011; Kim et al., 2014; Giraldo-Chica et al., 2018; Gong et al., 2019; Clark et al., 2020), autism spectrum disorders (Verly et al., 2014; Khan et al., 2015; Olivito et al., 2017; Hegarty et al., 2018), and addiction (Bora et al., 2012; Kühn et al., 2012; Moulton et al., 2014).

Likewise, changes in the BG and its networks result in motor abnormalities, cognitive dysfunction, and neurological symptoms (see reviews Ring, 2002; Marchand, 2010), highlighting the wide ranging impacts of this brain area. Parkinson's and Huntington's diseases are neurodegenerative disorders that affect dopaminergic and GABAergic neurons in the BG, respectively, and are associated with functional connectivity changes within the BG (Unschuld et al., 2012; Rolinski et al., 2015; Gargouri et al., 2016; Fritze et al., 2021). The BG are also associated with psychiatric symptoms. In those with sub-threshold depression, decreased globus pallidus volumes were related to scores on a depression inventory (Li et al., 2017) and those with generalized anxiety disorder, had less dopamine transport availability, leading to less dopamine in the striatum (Lee et al., 2015). Additionally, patients with schizophrenia and schizoaffective disorders displayed increased putamen volume relative to healthy controls (HC; Fritze et al., 2021), while striatal volume (Mittal et al., 2010) and motor deficits were associated with the conversion to psychosis in clinically-at-risk individuals (Mittal et al., 2010; Masucci et al., 2018). Lastly, the region has been associated with mental illnesses like addiction and substance abuse disorders, indicating changes in salience that affect approach behavior and motivation (see review Belin et al., 2009).

Increasingly, pathology and cognitive deficiencies linked to discrete CB, BG, or cortical regions have instead been suggested to be related to dysregulated network connectivity within and between the CB, BG, and the cortex. The CB and BG communicate with the cortex *via* discrete, trans-thalamic loops (reviewed in Kelly and Strick, 2003, 2004; Di Martino et al., 2008; Bostan and Strick, 2018), and with each other indirectly through these cortical projections. However, the BG and CB also communicate directly through subcortical pathways. Originally described in non-human primates, disynaptic anatomical pathways connect the dentate nucleus of the CB to the striatum of the BG (Hoshi et al., 2005) *via* the thalamus, and connect the subthalamic nucleus of the BG to multiple regions of the CB *via* the pontine nucleus (Bostan et al., 2010). These anatomical connections give credence to the concept of both cortical modulation by the CB and BG and of subcortical functional interconnectivity.

The thalamus, a region through which the CB and BG communicate with the cerebral cortex, was found to have structural differences, such as reduced volume in patients with schizophrenia (Huang et al., 2017), anxiety (Wang et al., 2018; Modi et al., 2019; Carey et al., 2021), depression (Li et al., 2017), Parkinson's Disease (Carey et al., 2021) and reduced microstructural complexity in first-episode psychosis (Huang et al., 2017; Cho et al., 2019). Thalamic functional connectivity differences have also been found in patients with schizophrenia and schizoaffective disorders (Huang et al., 2017; Ferri et al., 2018; Cho et al., 2019; Gong et al., 2019; Zhao et al., 2019; Fritze et al., 2021), and in obsessive-compulsive disorder (Haynes et al., 2018). Aberrant thalamic-putamen functional connectivity was found in those with unipolar depression (Marchand et al., 2012) and aberrant functional and anatomical connectivity between the BG and cortex has been noted in those with schizophrenia (Zhao et al., 2019). Because the CB and BG, as well as regions along the pathway enabling cortical communication, have been implicated in a wide range of psychiatric and neurological disorders, a network-level approach may be helpful in identifying differences in brain activation and connectivity patterns for our understanding of subcortical network contributions to behavior as well as symptomatology. As there is variability in behavior across individuals, including experiences of symptoms associated with diagnoses like anxiety and depression (at subclinical levels and in the absence of a formal diagnosis), using large community samples can provide important new insights into the associations between CB-BG connectivity, as well as variability in behavior and experiences with symptoms often associated with psychopathology.

Here, we were interested in exploring whether CCBN and MCBN GE is related to psychiatric symptomatology and cognitive task scores in a community sample selected from the Human Connectome Project (HCP) 1200 release. We hypothesized that CCBN GE would predict scores on self-reported anxiety, depression, and intrusive thoughts and cognitive tasks (processing speed, list sort, card sort, and flankers). We also hypothesized that the MCBN GE would predict scores on cognitive tasks and motor-associated psychiatric symptomology (hyperactivity, inactivity). Additionally, due to associations between working memory tasks and motor systems (Balsters et al., 2013; Von der Gablentz et al., 2015; Bernard et al., 2020; Clark et al., 2020), we hypothesized MCBN GE would predict scores on working memory tasks. Additionally, we previously found CB-BG GE is associated with the GE of canonical resting-state networks in a sample with no history of mental illness, illicit drug use, or alcohol and marijuana abuse (Jackson and Bernard, 2022). Here, we looked to replicate and extend these findings by broadening the selection criteria to include all subjects in the HCP 1200 release and by using a regression model to allow for the control of other potential explanatory variables such as alcohol and drug abuse. Lastly, given the underlying hypothesis that the CB-BG networks work in conjunction with cortical networks to affect behavior (Bostan and Strick, 2018), we also explored whether entering both CB-BG network and cortical network GE into a regression simultaneously predicts cognitive and symptom metrics.

## Method

### Participants—HCP 1200

All right-handed participants with a complete set of preprocessed resting-state functional MRI (rsfMRI) data with no noted issues in quality assurance from the S1200 Release of the Human Connectome Project (WU-UMN HCP Consortium) were selected for inclusion in this study (*N* = 783). A complete set of images was defined as one T1w structural image and four 15-min resting-state fMRI sessions (total of 1 h), each with associated within-subject 6-axis motion correction regressors. Outliers (defined as having a standard deviation of >3) for each analysis were removed and thus each analysis contains a slightly different number of participants. The degrees of freedom for each test are listed in Supplementary Tables 2, 3.

### HCP data acquisition and preprocessing

rsfMRI data for each participant consisted of four 15-min runs collected over 2 sessions [1,200 volumes, 720 ms TR, 2 mm isotropic voxels; see Van Essen et al. (2012) for full details on fMRI acquisition]. All anatomical and functional data were collected on a 3T HCP Siemens Skyra “Connectom” scanner. Behavioral data, preprocessed T1w structural images, rsfMRI functional images, and motion regressor data were downloaded from the HCP S1200 Release Amazon Web Services repository. Details of the preprocessing pipeline for functional images are fully described in Glasser et al. (2013). Structural scans had undergone gradient distortion correction, bias field correction, and registration to the 0.8 mm resolution MNI brain using Multimodal Surface Matching (Glasser et al., 2013, 2016). Additionally, structural images underwent tissue type segmentation and functional images underwent smoothing (5 mm FWHM) and artifact detection (global signal z-value threshold: 5, subject motion threshold: 0.9 mm) using the CONN toolbox (v. 20b; Whitfield-Gabrieli and Nieto-Castanon, 2012), a Matlab-based application designed for functional connectivity analysis. CONN was used with MATALB R2020a on centOS 7. Data were then denoised using confound regressors for 5 temporal components each from the segmented CSF and white matter, 24 motion realignment parameters, signal and/or motion outliers, and the 1st order derivative from the effect of rest. Finally, data underwent linear detrending and bandpass filtering (0.008–0.09 Hz). No difference in resting-state ROI-to-ROI functional connectivity was found between day 1 and 2 of data collection (*ps* > 0.5).

### Regions of interest

Using fslmaths, 3.5 mm spherical, binarized regions of interest (ROIs) representing two rsfMRI networks of CB and BG nodes (cognitive and motor CB-BG network), and 7 rsfMRI cortical networks [3 cognitive-associated: frontal-parietal network (FPN), cingulo-operculum network (CON), and default mode network (DMN); 1 motor-associated: motor network (MN); 1 emotion-associated: emotional network (EN); and two sensory-associated: visual network (VN), and auditory network (AN)] were created using fslmaths (FSL v.6.0.3: FMRIB's Software Library, www.fmrib.ox.ac.uk/fsl; Jenkinson et al., 2012).

CB-BG network ROIs were created using CB and BG coordinates previously reported on (Hausman et al., 2019). Lobule V and Lobule VI of the CB and dorsal caudal and dorsal rostral putamen of the BG were used to define the MCBN; while Crus I and Crus II of the CB and the inferior and superior ventral striatum, dorsal caudate, and ventral rostral putamen of the BG were used to define the CCBN. This division is in line with previous work parcellating each region based on resting-state and task-based functional connectivity and behavior (Balsters and Ranganath, 2008; Di Martino et al., 2008; Stoodley and Schmahmann, 2009; Salmi et al., 2010; Stoodley et al., 2012; Hausman et al., 2019; King et al., 2019). Importantly, Lobule VI, included in the MCBN, is known to also be related to cognitive processes related to spatial working memory and motor behavior (Schmahmann, 2019). Here, we were interested in the complex interplay between motor and cognitive processes and consider Lobule VI to be key in deciphering motor cognition from abstract cognition related to executive functioning. We similarly constructed cortical networks spanning cognitive, motor, emotional, and sensory domains. The cognitive networks were defined using ROIs from two task-positive rsfMRI networks, the cingulo-opercular (CON) and the fronto-parietal (FPN), and the default mode network (DMN), using coordinates originally reported by Fair et al. (2009). Motor network (MN) ROIs were created using coordinates based on Mayka et al. (2006). Emotional Network (EN) ROIs were created using coordinates originally reported by Stein et al. (2007) and adapted using information from Deen et al. (2011). The visual and auditory sensory networks, VN and AN, were defined using coordinates reported by Cassady et al. (2019). Importantly, given the association the CB and BG have with cortical and limbic networks, especially the MN, we did not use any CB or BG ROIs in any of the cortical networks. This required change of the VN only, for which we removed an ROI for CB Lobule VI. Removing this ROI allowed us to directly correlate measures from within the CB-BG networks to measures from within cortical networks. Additionally, to account for the use of both left and right ROIs in some cortical networks and for the unilateral nature of subcortical networks (Pelzer et al., 2017), all lateralized (defined as more than 3 mm from the midline in the x direction) ROIs without a reported contralateral match were mirrored in the x direction. Supplementary Table 1 provides details for the 122 resulting ROIs (for a visual depiction of their locations, please see Jackson and Bernard, 2022).

### Symptom and cognitive measures

For analyses including diagnostic symptom scales, we used the total scores for the Achenbach Self Report (ASR) for Intrusive thoughts, DSM Depressive, DSM Anxiety, DSM Inactivity, and DSM Hyperactivity measures. For analyses of cognitive function, we utilized scores from the NIH Toolbox for executive function (card sort, flankers), working memory (list sorting), and processing speed (pattern completion). Please see the HCP 1200 release manual for further detailed descriptions of these measures (https://www.humanconnectome.org/storage/app/media/documentation/s1200/HCP_S1200_Release_Reference_Manual.pdf). Each measure was centered and demeaned prior to entry into analyses.

### First-level FMRI analysis, global efficiency

All participant-level computations were performed in the CONN toolbox (v. 20b; Whitfield-Gabrieli and Nieto-Castanon, 2012). For each participant, a timeseries for each of the 122 ROIs was extracted and cross-correlated using bivariate analyses, resulting in a correlation matrix (122 × 122) for each participant. To compute graph theory components, edges were defined as thresholded correlation coefficients and nodes were defined as the ROIs in each network as described above (Stein et al., 2007; Fair et al., 2009; Geerligs et al., 2015; Hausman et al., 2019). We previously investigated differences in network measures when different thresholds were used and found no substantial differences; as such, we used β > 0.1 to define edges, a correlation coefficient threshold commonly used in the literature (i.e., Fair et al., 2009) and consistent with prior work (Jackson and Bernard, 2022). To define interconnectedness, network-level GE, a graph theory metric of how efficiently information in a network travels, was computed separately for each of the nine networks (Latora and Marchiori, 2001; Achard and Bullmore, 2007; Whitfield-Gabrieli and Nieto-Castanon, 2012; Sheffield et al., 2016).

### Exchangeability blocks and PALM

Some participants in this sample were members of the same family and thus have shared genetic and environmental variance. To account for this, we used Permutation Analysis of Linear Models (PALM v. 119a; https://fsl.fmrib.ox.ac.uk/fsl/fslwiki/PALM) to permute the data using exchangeability blocks designed to separately capture the unique variance within monozygotic twins, dizygotic twins, and non-twin siblings while simultaneously accounting for family structure (i.e., size and type of sibships within one family). To do this, we generated exchangeability blocks using the February 2017 version of the hcp2blocks script provided by the developers of PALM (retrieved from https://github.com/andersonwinkler/HCP). All analyses herein utilized this method.

### Analysis design

All analyses were conducted using PALM and thus accounted for within-family variance and variance associated with family structure (see above). Variance associated with age, drug, and alcohol use was controlled for by entering the following demeaned columns from the HCP restricted data file into the analyses as covariates: Age_in_Yrs, SSAGA_Alc_12_Frq (Alcohol), SSAGA_Times_Used_Cocaine, SSAGA_Times_Used_Hallucinogens, SSAGA_Times_Used_Opiates, SSAGA_Times_Used_Sedatives,SSAGA_Times_Used_Stimulants, and SSAGA_Mj_Times_Used (Marijuana). All independent and dependent variables and covariates were centered and demeaned prior to entry into analyses.

Cognitive or motor CB-BG network GE was entered as the independent variable for all analyses and the opposite network was used as an additional control variable to remove any shared variance between the two sub-networks. The dependent variable for each test was the GE for each canonical resting-state network, self-reported symptomology scores, or cognitive performance. Outliers, defined as 3 *SD* from the mean, were removed from each independent, dependent, and covariate measure and resulted in slightly different sample sizes between analyses (see Supplementary Tables 2, 3). Exchangeability blocks were recalculated accordingly. We were also interested in teasing apart subcortical and cortical contributions to the symptom and cognitive scores. Due to limitations in how PALM reports coefficients, we were unable to meaningfully enter all networks into a larger regression model. We instead explored regression models that included both a CB-BG network and a cortical network as independent variables predicting cognitive and symptom metrics, detailed in **Table 3**. All covariates detailed above were controlled for using the same methods in these analyses. All analyses were subjected to multiple comparison correction using FDR, accounting for *p*-values within each type of test (i.e., all GE-GE analyses; all behavioral analyses) as implemented by R's p.adjust function.

## Results

Table 1 contains the *t*-statistic and significance level of each regression predicting cognitive and symptom scores. Lower MCBG GE predicted higher scores on the ASR DSM depression and hyperactivity measures (*ps* < 0.05). No other significant relationships were found after multiple comparisons corrections (*ps* > 0.06); however, there is a marginal positive association between CCBG GE and hyperactivity scores (*p* = 0.06), suggesting a dissociation between the CCBG and MCBG networks and hyperactivity. See Supplementary Table 2 for degrees of freedom for each analysis.

**Table 1:** T-statistics detailing the relationship between CB-BG network GE and cognitive and symptom metrics.

	**Anxiety**	**Depression**	**Intrusive thoughts**	**Inactivity**	**Hyperactivity**	**Processing speed**	**List sort**	**Flankers**	**Card sort**
CCBG GE	0.16	1.38	0.83	−0.36	2.18	−0.1	1.07	0.26	−0.055
MCBG GE	−1.73	−2.28*	−1.52	−0.60	−2.18*	−1.62	−0.80	−1.79	−0.39

Each analysis contained a different number of participants due to outliers. Degrees of freedom for each test is listed in Supplementary Table 2; Each *p*-value is FDR corrected: ^*^p < 0.05.

Table 2 contains the *t*-statistic and significance level of each regression predicting cortical network GE. CCBG GE predicted the GE of each cortical network (*ps* < 0.05), except for the EN (*p* = 0.29); the strongest associations were with the DMN and AN (*ps* < 0.001). MCBG GE predicted the GE of each cortical network (*ps* < 0.05); the strongest associations were with the EN, MN, and AN (*ps* < 0.001). See Supplementary Table 3 for degrees of freedom for each analysis.

**Table 2:** T-statistics detailing the relationship between CB-BG network GE and that of each cortical network.

	**FPN**	**CON**	**DMN**	**EN**	**MN**	**VN**	**AN**
CCBG GE	2.51*	2.87*	4.35**	1.05	2.94*	3.30*	3.94**
MCBG GE	2.69*	2.77*	2.94*	4.58**	3.62**	2.74*	4.61**

Each analysis contained a different number of participants due to outliers. Degrees of freedom for each test is listed in Supplementary Table 3; Each *p*-value is FDR corrected: ^**^p < 0.001, ^*^p < 0.05.

**Table 3:** T-statistic of each exploratory regression model that included both a CB-BG network and a cortical network as independent variables predicting cognitive and symptom metrics.

***t*_(782)_**	**Anxiety**	**Depression**	**Intrusive thoughts**	**Inactivity**	**Hyperactivity**	**Processing speed**	**List sort**	**Flankers**	**Card sort**
CCBG-FPN	1.04	1.92	0.87	0.5	1.85	0.48	−0.75	0.95	−0.16
MCBG-FPN	−1.5	−2.08	−1.81	−0.76	−2.65	−1.73	1.99	−1.76	−0.34
CCBG-CON	0.32	0.86	0.38	0	2.38	−0.67	2.06	0.99	−0.85
MCBG-CON	−1.75	−2.37	−1.93	−0.89	−2.59	−1.99	−0.83	−1.8	−0.51
CCBG-DMN	0.8	1.56	2.38	1.52	3.27*	−0.19	2.82*	1.4	−0.57
MCBG-DMN	−1.57	−2.13	−1.37	−0.45	−2.23	−1.89	−0.48	−1.6	−0.45
CCBG-MN	−0.65	0.44	0.43	0	2.4	−0.58	2.19	0.75	0.39
MCBG-MN	−1.95	−2.43	−1.9	−0.89	−2.53	−1.98	−0.74	−1.82	−0.25
CCBG-EN	0.66	0.67	0	0.45	2.17	−0.57	2.05	1.13	0.15
MCBG-EN	−1.6	−2.32	−1.98	−0.75	−2.43	−1.99	−0.66	−1.66	−0.29
CCBG-AN	−0.76	0.42	0.95	−0.7	1.42	−0.18	3.11*	0.77	0.14
MCBG-AN	−1.9	−2.41	−1.82	−0.98	−2.74*	−1.89	−0.77	−1.86	−0.38
CCBG-VN	−0.74	0.46	0.46	−0.15	2.43	−0.83	4.05**	0.83	−0.35
MCBG-VN	−2.05	−2.34	−1.81	−0.91	−2.13	−2.11	0.37	−1.61	−0.38

Each *p*-value is FDR corrected: ^**^p < 0.001, ^*^p < 0.05.

Table 3 details the t-statistic and significance level of each exploratory regression model that included both a CB-BG network and a cortical network as independent variables predicting cognitive and symptom metrics. The model including CCBN and DMN GE positively predicted list sort scores (*p* = 0.04), as did CCBN and sensory network GE (AN: *p* = 0.03; VN: *p* < 0.001). Hyperactivity was positively predicted by the CCBN and DMN model (*p* = 0.02) and negatively predicted by the MCBN and AN model (*p* = 0.04), mirroring our main analyses.

## Discussion

Here, we used data from the Human Connectome Project 1200 release to investigate whether CB-BG network GE relates to scores on self-reported psychiatric symptomology and cognitive task performance after controlling for variance due to familial relationships and family structure, age, alcohol, marijuana, and illicit drug use, and variance shared with the non-predictor CB-BG network. We found that MCBN GE negatively predicted self-reported hyperactivity (Table 1), as we hypothesized. That is, low efficiency within the MCBN predicted higher scores on self-reported hyperactivity, consistent with previous work indicating hyperactivity-associated impaired resting-state network connectivity (McLeod et al., 2014; Gao et al., 2019). Hyperactivity is associated with a reduction in motor control, specifically motor inhibition (e.g., Lijffijt et al., 2005). This suggests that an inefficient MCBN may be related to or indicative of a potential decrease in motor inhibition. We did not find an association with self-reported inactivity however, suggesting the efficiency of CB-BG communication is not related to self-reported motivation.

We also found MCBN GE negatively predicted scores on a self-reported depression inventory, indicating lower MCBN GE is related to higher self-reported depression. While this was not hypothesized, this is in line with recent work in animal models that found CB neurons projecting to the BG *via* the dentate nucleus mediates depression symptoms (Baek et al., 2022). Interestingly, no significant findings were present (*p* > 0.09) when cortical network GE was included in the model, indicating the relationship with depression is best explained by MCBN GE. This work further supports the recent argument for including motor system within the Research Domain Criteria framework for depression (Walther et al., 2019). Those with depression display aberrant psychomotor agitation and retardation, as noted in the Diagnostic and Statistical Manual 5 (American Psychiatric Association, 1996). Aberrant functional connectivity between the CB and cortex (Ma et al., 2013) and between the BG and cortex (Furman et al., 2011) has also been noted in depression. Here, this novel finding suggests a subcortical network of CB and BG motor regions also predicted symptoms of depression. Finally, although we did not find that symptoms of depression were associated with cognitive CB-BG network measures, Yoshida et al. (2017) found that human resting-state functional connectivity between the supplementary motor area and the DMN, a network highly related to CCBN GE, was related to depression. This highlights important questions about the complex interplay between the cognitive and motor systems in clinical symptomology as they relate to functional organization.

Exploratory models including CCBN GE and both AN and VN GE were both significant predictors of list sort scores, highlighting the potential importance of these sensory networks in some domains of cognitive processing. No other psychiatric symptom or cognitive score was significantly associated with CB-BG GE after correcting for multiple comparisons. However, an interesting marginal (*p*_*FDR*_ = 0.06) result involving CCBN GE and hyperactivity is of note due to the potential implication of the findings and the significance level prior to correction (*p* = 0.03). We found that, while *low* MCBN GE predicted *high* scores on self-reported hyperactivity, *high* CCBN GE scores predicted *high* hyperactivity (Table 1). Importantly, our exploratory analyses provide additional evidence for considering this marginal finding. Mirroring the main GE analyses, we found that the model including CCBN and DMN *positively* predicted hyperactivity while the model including MCBN and AN *negatively* predicted hyperactivity. These findings taken together suggest dissociable relationships between subcortical motor and cognitive systems and hyperactivity wherein lower measures of network connectivity in motor control regions (evidenced by low MCBN GE) and higher measures in cognitive control regions (evidenced by high CCBN GE) differentially predict self-reported hyperactivity (albeit with the caveat of a marginal result). While not explicitly significant, we consider this inverse relationship between the cognitive and motor regions of the CB and BG to be worth noting for the potential identification of future clinical biomarkers. Indeed, previous research provides support for our interpretation. Kucyi et al. (2015) found CB regions associated with the DMN (Crus I and Crus II) exhibited higher functional connectivity with cortical DMN regions in those with attention-deficit hyperactivity disorder (ADHD), signifying the potential dysfunction of cognitive CB regions in ADHD. Similarly, Sörös et al. (2019) found an increase in functional connectivity of BG regions and the AN in those with ADHD, also in line with our exploratory analyses. Therefore, while our results fail to meet the threshold set for significance testing, we cautiously posit that the CB and BG may influence hyperactivity by regulating the inhibition of the DMN and that this mechanism may be disrupted in those suffering from disorders that present with hyperactivity. Indeed, taking all findings into account, symptoms of depression that present with agitation and hyperactivity may be facilitated by the cognitive CB-BG subcortical circuits. However, future research is warranted, to determine the replicability of these findings given the marginal effect, and to further identify the underlying dynamics of these regions and reported hyperactivity symptoms.

We also replicated and extended our prior work detailing correlations between CB-BG and cortical networks (Jackson and Bernard, 2022) by expanding the sample size 3-fold and introducing additional controls to probe the network relationships. We found CCBN GE strongly predicted DMN and AN GE, moderately predicted MN GE, and did not predict EN GE, largely consistent with our previous findings and suggestive of CCBN involvement in self-referential thought and auditory/verbal working memory (Fox et al., 2015). We additionally found CCBN GE predicted FPN, CON, and VN GE. In our initial work, we predicted a relationship between CCBN GE and both FPN and CON GE due to the cognitive nature of each of the networks, yet we only found marginal results. Here, our findings indicated that these networks may indeed be related, contrary to our original results. Lastly, recent findings suggest the CB may be involved with low-level sensorimotor systems (Brissenden et al., 2016; van Es et al., 2019). Though our previous study only found a link between MCBN GE and VN GE, we posit that the regions within the CCBN may also be involved and needed additional power to be detected. Additionally, no relationship was found between the CCBN and EN, replicating our previous report (Jackson and Bernard, 2022) in which we predicted and did not find an association due to the association between the EN and attentional processes (Arnsten and Rubia, 2012). This may be interpreted as the CB-BG communicating primarily with different regions of the CB and BG or perhaps simply that EN integration is not related to CCBN integration, regardless of connectivity. Future work investigating the relationship between EN nodes and limbic-associated nodes within the CB and BG may clarify this distinction.

Likewise, we largely replicated our previous findings that MCBN GE strongly predicts MN and AN GE; while only moderately predictive of FPN, CON, and VN GE. Additionally, we found that MCBN GE also strongly predicts EN and moderately predicts DMN GE. This was not predicted, but is not necessarily surprising, as the GE of each of these networks were marginally related to MCBN GE in our previous report (Jackson and Bernard, 2022) and significantly related when cost or degree (as opposed to GE) were used to describe networks, indicating these networks are, like the regions above, also related but require additional power or controls to detect. This replication advances our knowledge of cortical-subcortical network relationships and show the effects are relatively stable within both a selected, healthy sample and within a community sample consisting of non-selected participants displaying a range of drug behavior and mental health symptomology.

### Limitations

Here, we took advantage of a large, community sample to explore the relationships between CB-BG networks and scores on cognitive and psychiatric symptom measures. Of note, this sample and the sample in our previous report (Jackson and Bernard, 2022) are from the same larger dataset and share participants. This overlap between samples was seen as a strength, as it allowed us to compare changes due to our control methods (we previously used partial correlations to look at subcortical-cortical network relationships) and the inclusion of participants with self-reported drug use.

Another potentially significant limitation of these analyses is the exploratory and non-directional nature of our predictions. We however argue these analyses were a necessary first step in determining whether the CB-BG network was related to cognitive and psychiatric symptomology and will be useful to future researchers in the search for clinically relevant biomarkers. Relatedly, while we were well powered to detect effects, future work may seek to limit the scope and make specific predictions to limit any type II errors introduced because of multiple comparison corrections.

Additionally, here we were interested in whether the GE of CB-BG networks predicted cognitive and psychiatric symptom measures. GE was used as a proxy for intra-network integration, which can be conceptualized as the “interconnectedness” of the network. Thus, our findings here indicate that CB and BG communication patterns are predictive of symptoms of depression and hyperactivity scores but do not provide a causal link and must be interpreted cautiously. Of note, we relied on self-reported data for psychiatric symptomology, potentially decreasing the detectable signal. Future studies might address this by using data from structured diagnostic interviews to inform symptomology. We were also unable to investigate development differences in these networks as they relate to transdiagnostic psychiatric symptoms due to the limited age range of the sample (18-35). Future works look at how these networks develop and attempt to develop predisposition measures based on network interconnectivity.

Regarding network selection, our MCBN included CB Lobule VI, a posterior region known to be associated with motor cognition and spatial working memory (Schmahmann, 2019). While we chose this region to form a motor network that included nodes related to motor cognition (as opposed to strictly motor behavior), we recognize this could limit the interpretability of the MCBN's association with cognitive processes. Future studies might benefit from selecting other CB-BGnodes or by using a data-driven component analysis to determine network inclusion. Relatedly, we did not include a CB-BG network associated with limbic processes in our analyses. This was for two reasons. First, we chose the CCBN and MCBN nodes primarily due to their known interconnectivity, as reported in our previous work (Hausman et al., 2019; Jackson and Bernard, 2022). Second, we were concerned about detecting a reliable signal in the fastigial nuclei, CB regions we would have included, and how this additional noise may affect our analyses due to the inclusion of each CB-BG as a control.

The selected analysis tool, PALM, was chosen for the ability to permute data using exchangeability blocks, it did had several limitations. We were unable to see coefficients for the regression models and are therefore unable to attribute significant contributions to the model to any one predictor or covariate. We were also unable to create subject-level residuals to create scatterplots depicting the significant relationships.

Lastly, while not explicitly a limitation, we utilized functional imaging to create the correlation matrices we used to compute the GE of our networks and thus did not investigate the white matter structure of these networks using diffusion MRI. Previous reports found decreased posterior CB fractional anisotropy (FA) in patients related to depression inventory scores (Peng et al., 2013) and increased striatal FA in ADHD (Silk et al., 2009). Future studies should attempt to relate FA within regions of the CB-BG networks with depression and hyperactivity. We speculate differentiable contributions between cognitive and motor regions of the CB and BG in line with our main and marginal findings detailed above.

## Conclusion

Here, we demonstrated a relationship between motor CB-BG network and scores on self-reported depression and hyperactivity questionnaires. We also found cognitive CB-BG regions marginally predicted hyperactivity, indicating a potential dissociation between the motor and cognitive regions as they relate to hyperactivity. Additionally, we replicated and extended our previous research depicting the relationships between CB-BG network GE and cortical network GE, underlining the stability of these network relationships even within non-selected community samples after controlling for drug and alcohol use. These analyses support the hypotheses that the CB and BG each play a significant role in both cognitive and motor behavior, including psychiatric symptomology of depression and hyperactivity.

## Data availability statement

Publicly available datasets were analyzed in this study. This data can be found here: https://www.humanconnectome.org/study/hcp-young-adult/document/1200-subjects-data-release.

## Ethics statement

The studies involving human participants were reviewed and approved by Texas A&M University. The patients/participants provided their written informed consent to participate in this study.

## Author contributions

TJ processed and analyzed data. JB supervised this work and provided editorial services. Both authors contributed to the article and approved the submitted version.

## Funding

Internal support for TJ provided for by the Elizabeth A. Qualls '89 Endowed Fellowship; Additional support provided by National Institute on Aging, Grant/Award Number: R01AG064010-01; Data were provided (in part) by the Human Connectome Project, WU-Minn Consortium (Principal Investigators: David Van Essen and Kamil Ugurbil; 1U54MH091657) funded by the 16 NIH Institutes and Centers that support the NIH Blueprint for Neuroscience Research; and by the McDonnell Center for Systems Neuroscience at Washington University. This work was supported in part by the Texas Virtual Data Library (ViDaL) funded by the Texas A&M University Research Development Fund.

## Conflict of interest

The authors declare that the research was conducted in the absence of any commercial or financial relationships that could be construed as a potential conflict of interest.

## Publisher's note

All claims expressed in this article are solely those of the authors and do not necessarily represent those of their affiliated organizations, or those of the publisher, the editors and the reviewers. Any product that may be evaluated in this article, or claim that may be made by its manufacturer, is not guaranteed or endorsed by the publisher.
