# Supplementary material for: Cerebellar and basal ganglia motor network predicts trait depression and hyperactivity
Source: Front Behav Neurosci. 2022 Sep 16;16:953303. doi: 10.3389/fnbeh.2022.953303 (PMC9523104; doi:10.3389/fnbeh.2022.953303)
Supplement: Supplementary file 1 [file Data_Sheet_1.docx]

| *Supplemental Table 1*. Abbreviations, region names, MNI coordinates, network association, and source for each ROIs used in the analyses. All ROIs are 3.5mm, spherical, and binarized. ROIs with the source "*Mirrored"* were created using its contralateral pair. | | | | |
| --- | --- | --- | --- | --- |
| Abbreviation | Region | MNI (X, Y, Z) | Network | Source |
| L DCP | Left dorsal caudal putamen | -28, 1, 3 | MCB-BG | Hausman, Jackson et al., 2019 |
| R DCP | Right dorsal caudal putamen | 28, 1, 3 | MCB-BG | *Mirrored* |
| L DRP | Left dorsal rostral putamen | -25, 8, 6 | MCB-BG | Hausman, Jackson et al., 2019 |
| R DRP | Right dorsal rostral putamen | 25, 8, 6 | MCB-BG | *Mirrored* |
| R LobuleV | Right Cerebellar Lobule V | 16, -52, -17 | MCB-BG | Hausman, Jackson et al., 2019 |
| L LobuleV | Left Cerebellar Lobule V | -16, -52, -17 | MCB-BG | *Mirrored* |
| R LobuleVI | Right Cerebellar Lobule VI | 26, -60, -25 | MCB-BG | Hausman, Jackson et al., 2019 |
| L LobuleVI | Left Cerebellar Lobule VI | -26, -60, -25 | MCB-BG | *Mirrored* |
| L VSi | Left inferior ventral striatum | - 9, 9, -8 | CCB-BG | Hausman, Jackson et al., 2019 |
| R VSi | Right inferior ventral striatum | 9, 9, -8 | CCB-BG | *Mirrored* |
| L VSs | Left superior ventral striatum | -10, 15, 0 | CCB-BG | Hausman, Jackson et al., 2019 |
| R VSs | Right superior ventral striatum | 10, 15, 0 | CCB-BG | *Mirrored* |
| L DC | Left dorsal caudate | -13, 15, 9 | CCB-BG | Hausman, Jackson et al., 2019 |
| R DC | Right dorsal caudate | 13, 15, 9 | CCB-BG | *Mirrored* |
| L VRP | Left ventral rostral putamen | -20, 12, -3 | CCB-BG | Hausman, Jackson et al., 2019 |
| R VRP | Right ventral rostral putamen | 20, 12, -3 | CCB-BG | *Mirrored* |
| R CrusI | Right Cerebellar CrusI | 47, -61, -34 | CCB-BG | Hausman, Jackson et al., 2019 |
| L Crus I | Left Cerebellar CrusI | -47, -61, -34 | CCB-BG | *Mirrored* |
| R CrusII | Right Cerebellar CrusII | 41, -70, -46 | CCB-BG | Hausman, Jackson et al., 2019 |
| L CrusII | Left Cerebellar CrusII | -41, -70, -46 | CCB-BG | *Mirrored* |
| mCC | Medial cingulate cortex | 0, 29, 30 | FPN | Fair et al., 2009 |
| L dlPFC | Left dorsolateral prefrontal cortex | -43, 22, 34 | FPN | Fair et al., 2009 |
| R dlPFC | Right dorsolateral prefrontal cortex | 43, 22, 34 | FPN | Fair et al., 2009 |
| L FG | Left frontal gyrus | -41, 3, 36 | FPN | Fair et al., 2009 |
| R FG | Right frontal gyrus | 41, 3, 36 | FPN | Fair et al., 2009 |
| L IPL | Left inferior parietal lobule | -51, -51, 36 | FPN | Fair et al., 2009 |
| R IPL | Right inferior parietal lobule | 51, -47, 42 | FPN | Fair et al., 2009 |
| L IPS | Left intraparietal sulcus | -31, -59, 42 | FPN | Fair et al., 2009 |
| R IPS | Right intraparietal sulcus | 30, -61, 39 | FPN | Fair et al., 2009 |
| L Precun | Left precuneus | -9, -72, 37 | FPN | Fair et al., 2009 |
| R Precun | Right precuneus | 10, -69, 39 | FPN | Fair et al., 2009 |
| dACC | Dorsal anterior cingulate cortex | -1, 10, 46 | CON | Fair et al., 2009 |
| L aPFC | Left anterior prefrontal cortex | -28, 51, 1 | CON | Fair et al., 2009 |
| R aPFC | Right anterior prefrontal cortex | 27, 50, 23 | CON | Fair et al., 2009 |
| L aI | Left anterior insula | -35, 14, 5 | CON | Fair et al., 2009 |
| R aI | Right anterior insula | 36, 16, 4 | CON | Fair et al., 2009 |
| L aTh | Left anterior Thalamus | -12, -15, 7 | CON | Fair et al., 2009 |
| R aTh | Right anterior Thalamus | 10, -15, 8 | CON | Fair et al., 2009 |
| amPFC | Anterior medial prefrontal cortex | 1, 54, 21 | DMN | Fair et al., 2009 |
| vmPFC | Ventromedial prefrontal cortex | -3, 29, -2 | DMN | Fair et al., 2009 |
| pCC | Posterior cingulate cortex | -2, -36, 37 | DMN | Fair et al., 2009 |
| retsplen | Retro Splenial | 3, 51, 8 | DMN | Fair et al., 2009 |
| L SFC | Left superior frontal cortex | -14, 38, 52 | DMN | Fair et al., 2009 |
| R SFC | Right superior frontal cortex | 17, 37, 52 | DMN | Fair et al., 2009 |
| L ITL | Left inferior temporal lobe | -61, -33, -15 | DMN | Fair et al., 2009 |
| R ITL | Right inferior temporal lobe | 65, -17, -15 | DMN | Fair et al., 2009 |
| L Para(DMN) | Left parahippocampus | -22, -26, -16 | DMN | Fair et al., 2009 |
| R Para(DMN) | Right parahippocampus | 25, -26, -14 | DMN | Fair et al., 2009 |
| L lPC | Left lateral parietal cortex | -47, -67, 36 | DMN | Fair et al., 2009 |
| R lPC | Right lateral parietal cortex | 53, -67, 36 | DMN | Fair et al., 2009 |
| supCC | Supragenual cingulate | 0, 34, 30 | EN | Stein et al., 2007 |
| subCC | Subgenual cingulate | 0, 15, -14 | EN | Stein et al., 2007 |
| pCC | Posterior cingulate cortex | 0, -33, 38 | EN | Stein et al., 2007 |
| L OFC | Left orbitofrontal cortex | -46, 31, -9 | EN | Stein et al., 2007 |
| R OFC | Right orbitofrontal cortex | 46, 31, -9 | EN | *Mirrored* |
| L Para(EN) | Left parahippocampus gyrus | -26, -19, -14 | EN | Stein et al., 2007 |
| R Para(EN) | Right parahippocampus gyrus | 26, -19 -14 | EN | *Mirrored* |
| L lPFC | Left lateral prefrontal cortex | -56, 26, 25 | EN | Stein et al., 2007 |
| R lPFC | Right lateral prefrontal cortex | 56, 26, 25 | EN | *Mirrored* |
| L Amyg | Left amygdala | -26, 0, -20 | EN | Stein et al., 2007 |
| R Amyg | Right amygdala | 26, 0, -20 | EN | *Mirrored* |
| L vaI | Left ventral anterior insula | -33, 13, -7 | EN | Deen et al., 2011 |
| R vaI | Right ventral anterior insula | 32, 10, -6 | EN | Deen et al., 2011 |
| mPMC | Mesial prefrontal cortex | -3 -5 60 | MN | Mayka et al., 2006 |
| pSMA | pre-supplementary motor area | -4 3 59 | MN | Mayka et al., 2006 |
| SMA | Supplementary motor area | -3 -11 61 | MN | Mayka et al., 2006 |
| L lPMC | Left lateral premotor cortex | -26 -10 62 | MN | Mayka et al., 2006 |
| R lPMC | Right lateral premotor cortex | 25, -11, 61 | MN | *Mirrored* |
| L dlPMC | Left dorsolateral premotor cortex | -30 -8 64 | MN | Mayka et al., 2006 |
| R dlPMC | Right dorsolateral premotor cortex | 29 -9 64 | MN | *Mirrored* |
| L vlPMC | Left ventral lateral premotor cortex | -52 7 22 | MN | Mayka et al., 2006 |
| R vlPMC | Right ventral lateral premotor cortex | 52 6 21 | MN | *Mirrored* |
| L SMC | Left sensorimotor cortex | -39 -24 59 | MN | Mayka et al., 2006 |
| R SMC | Right sensorimotor cortex | 38 -25 59 | MN | *Mirrored* |
| L M1 | Left primary motor cortex | -37 -25 64 | MN | Mayka et al., 2006 |
| R M1 | Right primary motor cortex | 36 -21 58 | MN | *Mirrored* |
| L S1 | Left primary sensorimotor cortex | -40 -27 54 | MN | Mayka et al., 2006 |
| R S1 | Right primary sensorimotor cortex | 39 -28 54 | MN | *Mirrored* |
| L Ling | Lingual_L | -15, -72, -8 | VN | Cassady et al., 2019 |
| L Ling | Lingual_L | -16, -52, -1 | VN | Cassady et al., 2019 |
| R Ling | Lingual_R | 18, -47, -10 | VN | Cassady et al., 2019 |
| R Ling | Lingual_R | 20, -66, 2 | VN | Cassady et al., 2019 |
| R Ling | Lingual_R | 20, -86, -2 | VN | Cassady et al., 2019 |
| L Cal | Calcarine_L | -18, -68, 5 | VN | Cassady et al., 2019 |
| L Cal | Calcarine_L | -8, -81, 7 | VN | Cassady et al., 2019 |
| R Cal | Calcarine_R | 8, -72, 11 | VN | Cassady et al., 2019 |
| R Cal | Calcarine_R | 6, -81, 6 | VN | Cassady et al., 2019 |
| L Cun | Cuneus_L | -16, -77, 34 | VN | Cassady et al., 2019 |
| L Cun | Cuneus_L | -3, -81, 21 | VN | Cassady et al., 2019 |
| R Cun | Cuneus_R | 15, -77, 31 | VN | Cassady et al., 2019 |
| R Cun | Cuneus_R | 6, -72, 24 | VN | Cassady et al., 2019 |
| L Fus | Fusiform_L | -33, -79, -13 | VN | Cassady et al., 2019 |
| R Fus | Fusiform_R | 27, -59, -9 | VN | Cassady et al., 2019 |
| L SupOcc | Occipital_Sup_L | -14, -91, 31 | VN | Cassady et al., 2019 |
| R SupOcc | Occipital_Sup_R | 15, -87, 37 | VN | Cassady et al., 2019 |
| R SupOcc | Occipital_Sup_R | 24, -87, 24 | VN | Cassady et al., 2019 |
| L MidOcc | Occipital_Mid_L | -40, -88, -6 | VN | Cassady et al., 2019 |
| L MidOcc | Occipital_Mid_L | -28, -79, 19 | VN | Cassady et al., 2019 |
| L MidOcc | Occipital_Mid_L | -24, -91, 19 | VN | Cassady et al., 2019 |
| L MidOcc | Occipital_Mid_L | -42, -74, 0 | VN | Cassady et al., 2019 |
| L MidOcc | Occipital_Mid_L | -26, -90, 3 | VN | Cassady et al., 2019 |
| R MidOcc | Occipital_Mid_R | 40, -72, 14 | VN | Cassady et al., 2019 |
| R MidOcc | Occipital_Mid_R | 29, -77, 25 | VN | Cassady et al., 2019 |
| R MidOcc | Occipital_Mid_R | 37, -81, 1 | VN | Cassady et al., 2019 |
| R MidOcc | Occipital_Mid_R | 37, -84, 13 | VN | Cassady et al., 2019 |
| L InfOcc | Occipital_Inf_L | -47, -76, -10 | VN | Cassady et al., 2019 |
| R InfOcc | Occipital_Inf_R | 43, -78, -12 | VN | Cassady et al., 2019 |
| L InfTemp | Temporal_Inf_L | -42, -76, -10 | VN | *Mirrored* |
| R InfTemp | Temporal_Inf_R | 42, -66, -8 | VN | Cassady et al., 2019 |
| L Hes | Heschl_L | -30, -27, 12 | AN | Cassady et al., 2019 |
| L Hes | Heschl_L | -55, -9, 12 | AN | Cassady et al., 2019 |
| R Hes | Heschl_R | 32, -26, 13 | AN | Cassady et al., 2019 |
| L Sup Temp | Temporal_Sup_L | -60, -25, 14 | AN | Cassady et al., 2019 |
| L Sup Temp | Temporal_Sup_L | -49, -26, 5 | AN | Cassady et al., 2019 |
| R Sup Temp | Temporal_Sup_R | 65, -33, 20 | AN | Cassady et al., 2019 |
| R Sup Temp | Temporal_Sup_R | 58, -16, 7 | AN | Cassady et al., 2019 |
| L RolOp | Rolandic_Oper_L | -38, -33, 17 | AN | Cassady et al., 2019 |
| R RolOp | Rolandic_Oper_R | 43, -23, 20 | AN | Cassady et al., 2019 |
| R RolOp | Rolandic_Oper_R | 56, -5, 13 | AN | Cassady et al., 2019 |
| L SupMar | SupraMarginal_L | -50, -34, 26 | AN | Cassady et al., 2019 |
| L SupMar | SupraMarginal_L | -53, -22, 23 | AN | Cassady et al., 2019 |
| R SupMar | SupraMarginal_R | 59, -17, 29 | AN | Cassady et al., 2019 |

| Supplemental Table 2. Degrees of freedom for each test listed in Table 1. | | | | | | | | | |  |
| --- | --- | --- | --- | --- | --- | --- | --- | --- | --- | --- |
|  | Anxiety | Depression | Intrusive Thoughts | Inactivity | Hyperactivity | Processing Speed | List Sort | Flankers | Card Sort | |
| CCBG GE | 760 | 757 | 759 | 762 | 763 | 766 | 767 | 767 | 765 | |
| MCBG GE | 762 | 759 | 761 | 764 | 765 | 768 | 769 | 769 | 768 | |
| *Note: Each analysis contained a different number of participants due to outliers.* | | | | | | | | | |  |

| Supplemental Table 3. Degrees of freedom for each test listed in Table 1. | | | | | | | |
| --- | --- | --- | --- | --- | --- | --- | --- |
|  | FPN | CON | DMN | EN | MN | VN | AN |
| CCBG GE | 768 | 768 | 766 | 759 | 759 | 760 | 768 |
| MCBG GE | 769 | 769 | 767 | 760 | 760 | 761 | 769 |
| *Note: Each analysis contained a different number of participants due to removal of outliers.* | | | | | | | |
